# GLOBAL PUBLISHING OPPORTUNITIES IN GERONTOLOGY: A VIEW FROM THE EDITORS' DESKS

**DOI:** 10.1093/geroni/igz038.702

**Published:** 2019-11-08

**Authors:** Edward A Miller, Elizabeth Simpson, Michael Gusmano

**Affiliations:** University of Massachusetts Boston, Boston, Massachusetts, United States

## Abstract

Global aging has proceeded at an unprecedented and accelerating rate. The aging of the population creates both opportunities and challenges for elders, their families, and society in general. Importantly, there is substantial variation in the effects of and response to global aging both within and across nations depending, in part, on prevailing cultural expectations and values, political and economic imperatives, and social and demographic characteristics. Thus, while some regions and countries have responded with innovative policies and programs to better enable the growing cohort of older adults to remain active and engaged in the community, other regions and countries have struggled with their response or barely begun to plan for the rising population of elders. This symposium assembles editors at five leading gerontological journals to demonstrate the role that peer-reviewed scholarship can play in disseminating knowledge that informs gerontological research, policy, and practice internationally. Editors include: Jeffrey Burr, PhD, Research on Aging; Deborah Carr, PhD, Journal of Gerontology: Social Sciences; Edward Alan Miller, PhD, Journal of Aging & Social Policy; Julie Hicks Patrick, PhD, International Journal of Aging & Human Development; and Julie Robison, PhD, The Journal of Applied Gerontology. Each presenter will review the scope, content, and focus of their journals and the role and opportunities for international scholarship. Michael Gusmano, PhD, a leading expert on the economic, political, and social consequences of global aging and International Editor of the Journal of Aging & Social Policy, will serve as discussant.

